# Characterization of Biofilm Formation by the Dermatophyte *Nannizzia gypsea*

**DOI:** 10.3390/jof11060455

**Published:** 2025-06-14

**Authors:** Bruno B. A. Arantes, Ana Karla L. F. Cabral, Kelvin S. dos Santos, Matheus B. Mendonça, Rafaela C. dos Santos, Beatriz C. M. Bugalho, Lígia De S. Fernandes, Luis R. Martinez, Ana Marisa Fusco-Almeida, Maria José S. Mendes-Giannini

**Affiliations:** 1 Department of Clinical Analysis, School of Pharmaceutical Sciences, São Paulo State University UNESP, Araraquara 14800-903, Brazil; bruno.bulgarelli@unesp.br (B.B.A.A.); anakarlalfc@ufam.edu.br (A.K.L.F.C.); k.santos@unesp.br (K.S.d.S.); rafaela.cristine@unesp.br (R.C.d.S.); ana.marisa@unesp.br (A.M.F.-A.); 2 Laboratory of Medical Mycology, School of Pharmaceutical Sciences, Federal University of Amazonas-UFAM, Manaus 69077-000, Brazil; 3 Department of Oral Biology, College of Dentistry, University of Florida, Gainesville, FL 32610, USA; lmartinez@dental.ufl.edu; 4 Emerging Pathogens Institute, University of Florida, Gainesville, FL 32610, USA; 5 McKnight Brain Institute, University of Florida, Gainesville, FL 32610, USA; 6 Center for Translational Research in Neurodegenerative Disease, University of Florida, Gainesville, FL 32610, USA; 7 Center for Immunology and Transplantation, University of Florida, Gainesville, FL 32610, USA

**Keywords:** dermatophytes, *Nannizzia gypsea*, biofilms, extracellular matrix

## Abstract

Dermatophytosis is a fungal infection that affects the skin, hair, and nails, impacting approximately 25% of the global population. *Nannizzia gypsea* is a geophilic fungus that can cause infections in humans and animals. Several studies have been conducted regarding its virulence, or ability to cause disease. This species may produce keratinolytic enzymes and form biofilms, which can increase resistance to treatment. Thus, this study focuses on investigating the biofilm formation of *N. gypsea* isolated from canine dermatophytosis using an ex vivo hair model, its biofilm extracellular matrix macromolecular contents, and the expression of genes involved in the colonization of keratinized surfaces. The biofilm was analyzed for metabolic activity using the XTT reduction assay, crystal violet staining to measure biofilm biomass, scanning electron microscopy (SEM), and the presence of polysaccharides, proteins, and extracellular DNA in the biofilm extracellular matrix. The virulence genes subtilisin 7, fungalysin (extracellular metalloproteinase), and efflux pump (Multidrug and Toxin Extrusion Protein 2) were evaluated by qPCR, comparing the planktonic and biofilm phenotypes. *N. gypsea* formed a robust biofilm, which matured after 5 days. Scanning electron microscopy (SEM) revealed the presence of an extensive extracellular matrix. In the hair model, the characteristic ectothrix parasitism of the species is observable. The gene expression analysis revealed a higher expression of all evaluated genes in the biofilm form compared to the planktonic form. Thus, *N. gypsea* exhibits a biofilm characterized by a robust extracellular matrix and high gene expression of factors related to pathogenesis and resistance.

## 1. Introduction

Dermatophytosis, commonly known as tinea, is a fungal infection caused by filamentous fungi that has a strong affinity for keratinized structures, including skin, hair, and nails. It affects approximately 25% of the global population [1,2,3]. The rising incidence of this superficial mycosis, coupled with the emergence of antifungal resistance, underscores the need to understand its pathogenesis [4]. Previously classified into only three genera (*Microsporum*, *Trichophyton*, and *Epidermophyton*), dermatophytes are the causative agents of dermatophytosis worldwide. Phylogenetic analyses have now reorganized these fungi into new genera and species, including *Trichophyton*, *Epidermophyton*, *Nannizzia*, *Microsporum*, *Lophophyton*, *Paraphyton*, and *Arthroderma* [5,6]. *Nannizzia gypsea*, previously known as *Microsporum gypseum*, is geophilic and has attracted the attention of physicians, veterinarians, and epidemiologists due to its remarkable ability to infect dogs, cats, and other animals, as well as humans, with soil contact being an important means of dermatophytosis dissemination [7]. Lesions caused by *N. gypsea* can lead to alopecia, attributed to the fungus’s ability to produce proteolytic and keratolytic enzymes that degrade keratin, facilitating colonization of the stratum corneum [8] or the outermost layer of the skin.

Dermatophytes possess various virulence factors, and their ability to form biofilms is associated with resistance to antifungal treatments [9,10,11],^,^ which may contribute to the high recurrence rates of dermatophytosis [12,13]. A biofilm is a structured community of cells adhered to a surface and embedded in an extracellular matrix composed of polymeric substances. The advantages of biofilm formation for an organism include protection, nutrition, and survival in unfavorable environmental conditions, where it is constantly under pressure from both the immune system and antimicrobial agents [9,10,14,15]. Previous studies have demonstrated the ability of *N. gypsea* to form more robust biofilms compared to other species, such as *Trichophyton rubrum* and *Microsporum canis* [16]. The qualitative and quantitative characterization of biofilms and their transport mechanisms from the intracellular to the extracellular environment is crucial for clinical research. Once their structure, composition, or transport is elucidated, developing new drugs capable of preventing biofilm formation or making them permeable to existing drugs on the market becomes possible.

Several virulence factors have been described in dermatophytes, including adhesins that promote attachment to the stratum corneum; subtilisins, fungalysins, or metalloproteases specialized in keratin digestion; hemolysins, exotoxins capable of lysing red blood cells; phospholipases, which mediate the breakdown of membrane phospholipids in eukaryotic cells; and efflux pumps, proteins that transport drugs and potentially harmful molecules out of the fungal cell [17,18,19,20,21]. Secreted keratinases are mainly serine proteases and metalloproteases. The former belongs to the subtilisin subfamily (S8A), comprising 12 genes, SUB1 to SUB12, while the latter are classified into two distinct families: deuterolysin (M35) and fungalysin (M36). In addition to enzymatic virulence factors, some dermatophyte species exhibit non-enzymatic virulence factors associated with pathogenesis. For example, *Trichophyton rubrum* is known to produce xanthomegnin, a mycotoxin with immunosuppressive activity already reported in the literature [22,23]. Various experimental models have been proposed for the study of dermatophytes, including monolayer and spheroid cell cultures, as well as ex vivo models using nail and hair samples [24,25,26]. Thus, this study further characterizes biofilm formation by *N. gypsea* using an ex vivo hair model, scanning electron microscopy (SEM), relative gene expression analysis via real-time PCR, and determination of the biofilm’s extracellular matrix composition. We aim to understand *N. gypsea* colonization of and persistence in biotic surfaces to gain insight into dermatophytosis caused by this fungus and to develop therapeutics for its prevention, management, and treatment.

## 2. Materials and Methods

### 2.1. Microorganism

This study used a clinical isolate of *N. gypsea*, which was obtained from a canine dermatophytosis sample and deposited in the culture collection of the Clinical Mycology Laboratory/Proteomics Core of the School of Pharmaceutical Sciences of the Sao Paulo State University (UNESP). According to the caregiver’s report and the attending veterinarian’s evaluation, the dog presented symptoms of alopecia, erythema, pruritus, and desquamation, all clinical signs characteristic of dermatophytosis. At the time of isolation, the patient had not received any treatment, and the prescribed treatment was oral fluconazole at a dosage of 10 mg/kg. The isolate was grown and maintained on Sabouraud dextrose agar with chloramphenicol and incubated at 28 °C for 14 days. For the experimental assays, *N. gypsea* was further cultivated on malt extract agar and maintained under the same incubation conditions described. Since we used a clinical strain of *N. gypsea*, we validated its identification using ribosomal protein mass spectrometry using a MALDI-TOF mass spectrometry (MS) device (MALDI Biotyper^®^ Sirius Bruker, Billerica, MA, USA) from the Max Feffer Laboratory at the “Luiz de Queiroz” College of Agriculture (ESALQ, São Paulo, Brazil), made available by the supervising professor, Dr. Carlos Alberto Labate. Protein extraction was performed using protocols established by the manufacturer. The generated spectrum is compared with a reference database, allowing for identification at the genus or species level using Bruker Daltonics MALDI Biotyper software (version 3.0). In this identification, a log value is obtained to ensure the reliability of the result; therefore, only results with a log ≥ 2.0 are considered highly reliable and an identification (Figure S1). The equipment was previously calibrated with a mixture of proteins called BTS, as recommended and supplied by the manufacturer. The generated spectrum was not intended for detailed proteomic profiling but rather for microorganism identification based on the fingerprint of ribosomal and membrane proteins.

As complementary methods for the identification of the fungus, biochemical assays were also employed, including tests for hemolytic, urease, lipase, and protease activity. The following microorganisms were used as controls in these assays: *Streptococcus agalactiae* ATCC 13813 (β-hemolytic), *Staphylococcus aureus* ATCC 25923 (catalase positive), *Pseudomonas aeruginosa* ATCC 27853 (lipase positive), *Cryptococcus neoformans* ATCC 90112 (urease positive), and *Candida albicans* ATCC 90028 (protease positive). For hemolytic activity, blood agar was prepared using BHI agar supplemented with 5% defibrinated blood; urea agar was prepared by supplementing urea base agar with 40% urea solution; protease and lipase agar was prepared as described by Elavarashi et al. (2017) [17]. In addition, an antifungal susceptibility assay was performed using terbinafine and fluconazole. The antifungal susceptibility assay was conducted according to the guidelines of the Clinical and Laboratory Standards Institute (CLSI) [27], using a concentration range of 0.004 to 2.056 µg/mL for terbinafine and 0.125 to 64 µg/mL for fluconazole.

### 2.2. Biofilm Formation

After a seven-day cultivation of *N. gypsea*, a saline solution was added to the culture, and the fungal biomass was scraped and collected into a 15 mL conical tube. The suspension was homogenized using a vortex mixer, and the hyphae were allowed to settle for 2 min to separate and obtain conidia. After settling, the supernatant was carefully collected and transferred to another conical tube. A 1:10 dilution of this supernatant aliquot was prepared for counting in a hemocytometer, and the inoculum concentration was adjusted to 10^6^ cells/mL in saline solution [9,16,28].

A pre-adhesion step was performed by adding 200 μL of the standardized inoculum to each well of a 96-well microplate (Kasvi) and incubating at 37 °C for 4 h. After this period, the initial inoculum was removed, and each well was washed once with saline solution to remove non-adherent cells. After washing, 200 μL of Roswell Park Memorial Institute 1640 Medium (RPMI 1640, Sigma-Aldrich, St. Louis, MI, USA), buffered with 3-(N-morpholino) propanesulfonic acid (MOPS, Sigma-Aldrich) and supplemented with 2% glucose and L-glutamine, was added to each well. The microplate was incubated at 37 °C for 7 days, after which it was analyzed, with parameters evaluated every 24 h [9,28].

### 2.3. Biofilm Quantification

#### 2.3.1. XTT Reduction Assay

The metabolic activity of the biofilm was evaluated using the XTT (2,3-bis-(2-methoxy-4-nitro-5-sulfonylphenyl)-2H-tetrazolium-5-carboxanilide) reduction assay (Thermo Fisher Scientific, Waltham, MA, USA). Culture media were removed from the wells, and 54 µL of a solution containing XTT and 1 mM menadione was added to each well. The plates were sealed, protected from light, and incubated at 37 °C for 3 h. After this period, the optical density (OD) was measured at 490 nm using a spectrophotometer [9,28].

#### 2.3.2. Crystal Violet Staining

The culture medium was removed from each well containing the biofilm and washed twice with PBS for crystal violet staining, which stains both the cellular and matrix components of the biofilm. After drying at room temperature (RT), 200 µL of a 0.5% crystal violet solution in PBS was added to the wells for 20 min, then removed, and the wells were washed four times with water or until excess staining was removed. The biofilms were then decolorized by adding 200 µL of a 95% ethanol solution, and the wells were homogenized until the crystal violet staining was completely solubilized. The contents were transferred to a new microplate for spectrophotometric reading at a wavelength of 570 nm. This procedure was repeated at 1, 2, 3, 4, 5, and 6 days after biofilm formation [9,28].

### 2.4. Quantification of Extracellular Matrix Components

The extracellular matrix components of the *N. gypsea* biofilm were analyzed by cultivating the fungus in 24-well microplates using the same methodology described previously. The extracellular matrix was extracted by scraping the bottom of each well with sterile water and transferring the material to a conical tube. Protease inhibitors phenylmethanesulfonyl fluoride (PMSF, Thermo Fisher Scientific) and Leupeptin, Aprotinin, Antipain, and Chymostatin (LAAC, Thermo Fisher Scientific) were added to the samples at concentrations of 100 mM, using proportions of 10 μL/mL and 1 μL/mL, respectively. Following the addition of inhibitors, the suspensions containing the biofilms were sonicated at 40 kHz for 20 min. Subsequent assays quantified total proteins, total polysaccharides, and eDNA [29,30].

The quantification of total polysaccharides followed the method described by Dubois et al. (1956) [31]. In test tubes, 1.25 mL of concentrated sulfuric acid, 12.5 μL of 80% phenol solution, and 500 μL of the sample were added. After reagent addition, the tubes were incubated in a water bath at 37 °C for 20 min. The OD was measured at 485 nm, and polysaccharides were quantified based on a glucose standard curve (0–200 µg/mL).

The protein content was determined using the BCA Protein Assay Kit (Thermo Fisher Scientific), with optical density (OD) measured at 562 nm. Quantification was based on a bovine albumin standard curve (0–2000 μg/mL).

Extracellular DNA (eDNA) was extracted using the phenol-chloroform–isoamyl alcohol method. In a microtube, 500 μL of the sample and 500 μL of the phenol–chloroform–isoamyl alcohol solution (25:24:1) (Invitrogen) were added and homogenized by vortexing. The solution was centrifuged at 14,000 rpm for 15 min at 4 °C to separate the organic and aqueous phases. The supernatant was transferred to a new microtube, and chilled isopropanol (1:1) was added. The sample was then homogenized and incubated at −20 °C for 1 h to facilitate precipitation. After incubation, the solution was centrifuged at 14,000 rpm for 15 min at 4 °C. The supernatant was discarded, and the pellet was air-dried at RT. The genetic material was resuspended in 50 μL of 10 mM Tris-HCl buffer (pH 7.5) and 1 mM EDTA. EDNA was quantified by measuring absorbance at 260 nm [29].

### 2.5. Structural Analysis of Biofilms In Vitro and Ex Vivo by Scanning Electron Microscopy

For SEM analysis, the biofilm was cultivated in a 24-well microplate, as described. The biological material was fixed by removing all the culture medium and adding 800 μL of 2.5% (*v*/*v*) glutaraldehyde, followed by incubation at 4 °C for 1 h. After incubation, the fixative solution was removed, and the wells were air-dried at RT [32]. SEM analysis was conducted at the Scanning Electron Microscopy Laboratory of the Faculty of Dentistry at the UNESP Araraquara Campus, using a JEOL JSM-6610LV scanning electron microscope.

### 2.6. Hair Model Infection

A similar procedure to biofilm formation was performed using a hair model of infection. *N. gypsea* was scraped from the sporulation medium in sterile 0.85% saline and transferred to a 15 mL conical tube, vortexed to separate the mycelium from the conidia. The supernatant was collected, and the concentration was determined using a hematocytometer. For inoculum preparation, sterile saline was used to adjust the concentration to 10^6^ conidia/mL. In a 24-well microplate, discarded human beard hair, sterilized by autoclaving, was added, followed by the introduction of the inoculum. After a 4 h pre-adhesion step in saline solution, the medium was removed, and the RPMI-1640 medium, supplemented and buffered, was added under the same conditions as in the biofilm formation protocol [9,16].

### 2.7. Analysis of Virulence Gene Expression in N. gypsea

#### 2.7.1. Primer Design

Primers were designed based on FASTA sequences provided by the National Center for Biotechnology Information (NCBI) gene library, using OligoPerfect software (Thermo Fischer Scientific) for design (Table 1). The following parameters and values were used: primer size (19–21 bp); melting temperature (59–61 °C); GC content (50–60%); product size (80–150 bp); and a maximum number of G or C allowed in the last five 3′ bases (1–3 CGs). Then, OligoAnalyzer software (Integrated DNA Technologies) was used to verify physical parameters, such as melting temperature and hairpin temperature, using the following values: 50 mM Na⁺, 1.5 mM Mg^2^⁺, 0.2 mM dNTPs, and 0.5 μM primer. After primer design, the NCBI BLAST™ (TM Software 2.16.0+ version, Inc., Arcadia, CA, USA) tool from the National Institutes of Health (NIH) was used for in silico PCR reaction analysis to confirm specific annealing to *N. gypsea*. As complementary data, the amplicon size of each gene used in real-time PCR was analyzed. This analysis was performed using the in silico PCR tool from the Genomics Institute at the University of California, Santa Cruz (UCSC), and agarose gel electrophoresis.

#### 2.7.2. Evaluation of Genes Involved in Virulence and Pathogenicity by qPCR

Total RNA was extracted following the instructions of the Illustra RNAspin Isolation kit (GE Life Sciences, Marlborough, MA, USA). Concentration and purity were determined using a Nanodrop 2000 spectrophotometer (Thermo Scientific) at an optical density (OD) of 260 and 280 nm. The RNA integrity of the planktonic (Figure S2A) and biofilm (Figure S2B) samples used in real-time PCR reactions were evaluated. The analysis was performed using the Bioanalyzer 2100 system (Agilent Technologies, Santa Clara, CA, USA) according to the manufacturer’s specifications. Both sample types exhibited high purity and concentration. qPCR was conducted to assess the expression of subtilisin (Serine endoprotease, *Sub*7), fungalysin (extracellular elastolytic metalloproteinase, *Mmp12*), and efflux pump (Multidrug and Toxin Extrusion Protein 2, *Mate2*) genes in planktonic and biofilm-derived cells. Subsequently, 5 µg of total RNA was treated with the DNase I kit (Sigma-Aldrich), and complementary DNA (cDNA) was synthesized using the High-Capacity cDNA Reverse Transcription Kit (Applied Biosystems, Waltham, MA, USA) according to the manufacturer’s instructions. The qPCR was conducted using the 7500 Real-Time PCR System (Applied Biosystems, Waltham, MA, USA) at the Genomics Core Facility, Faculty of Pharmaceutical Sciences, at UNESP, Araraquara, with the detection system Power UP SYBR Green PCR Master Mix (Applied Biosystems). Relative expression was calculated using the 2⁻^ΔΔCt^ method, with glyceraldehyde-3-phosphate dehydrogenase (GADPH) as the endogenous control. Gene expression post-ex vivo infection was compared to in vitro results.

### 2.8. Statistical Analysis

All data were subjected to statistical analysis using Prism 10.1.4. (GraphPad Software, San Diego, CA, USA). *p*-values for individual or series of comparisons were calculated using paired two-tailed Student’s *t*-test or multiple Student’s *t*-test analyses, respectively. *p* < 0.05 was considered significant.

## 3. Results

### 3.1. N. gypsea Identification

We selected this clinical isolate for our studies based on the clinical presentation observed, its antifungal drug resistance profile, and the expression of virulence factors observed in preliminary tests. The literature highlights that the use of clinical strains may offer significant advantages, particularly when the objective is to investigate fungal–host interactions.

MALDI-TOF MS was used to identify this strain. The spectrum was analyzed by examining ribosomal proteins ranging from 2 to 20 kDa and compared with the database of the Bruker Daltonics MALDI Biotyper software (version 3.0). The Bruker Daltonics MALDI Biotyper software yielded a score of 2.11, confirming the identification of *N. gypsea*. As a result, molecular confirmation of the clinically isolated species used in this study was positively identified as *N. gypsea* F35 LLH (NCBI Identifier: 34384; Figure S1).

The strain *N. gypsea* F35 LLH was further characterized using qualitative biochemical tests and an antifungal susceptibility assay. *N. gypsea* F35 LLH showed catalase, lipase, protease, and urease activity (Table 2). However, the clinical isolate has no hemolytic activity. The minimum inhibitory concentration (MIC) obtained was ≥0.008 and ≥64 µg/mL for terbinafine and fluconazole, respectively.

### 3.2. Characterization of N. gypsea Biofilm Formation

**Figure 1:**
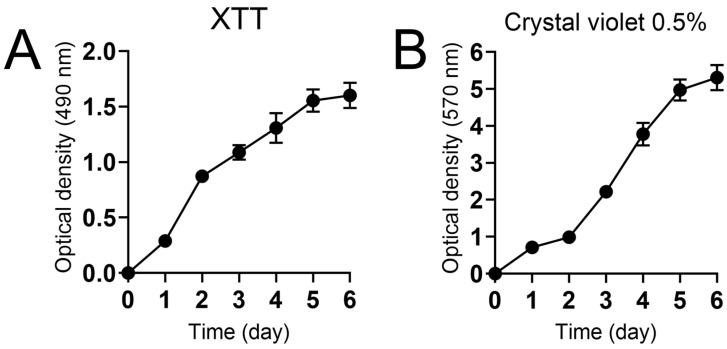
Characterization of *N. gypsea* biofilm formation in vitro. The (**A**) XTT reduction assay was performed to determine metabolic activity, and (**B**) crystal violet was used as a stain for biomass. Optical density (XTT: 490 nm and crystal violet: 570 nm) measurements were taken at specific time points. Each point denotes the average (*n* = 3) measurements. This experiment was conducted multiple times, yielding similar results each time.

We characterized the kinetics of *N. gypsea* biofilm formation over a six-day period (Figure 1). The metabolic activity of *N. gypsea* biofilm was determined using the XTT reduction assay, which showed a consistent hyperbolic increase after 5 days, followed by a plateau (Figure 1A). We then measured the *N. gypsea* biofilm biomass using the crystal violet staining method, which stains both cellular and extracellular matrix components. The biofilm biomass was found to increase significantly after 3 days of incubation (Figure 1B). Our results indicate that *N. gypsea* increases its cellular and extracellular matrix components over time, and its biofilm formation kinetics are like those previously described for filamentous molds.

### 3.3. The Macromolecular Composition of N. gypsea Biofilm Extracellular Matrix Increases over Time

**Figure 2:**
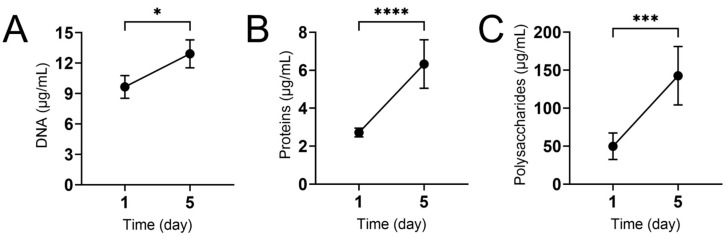
The content of macromolecules in the extracellular matrix of *N. gypsea* biofilms increases over time. We measured the concentration of (**A**) DNA, (**B**) proteins, and (**C**) polysaccharides in extracts of *N. gypsea* biofilm extracellular matrix at 1 and 5 days. Biofilms were incubated at 37 °C. Asterisks denote *p*-value significance (**** *p* < 0.0001; *** *p* < 0.001; * *p* < 0.05) calculated using Student’s *t*-test analysis. Each time point represents the average (*n* = 3) measurements. This experiment was conducted multiple times, yielding similar results each time.

Given the importance of the extracellular matrix in protecting fungal biofilms against environmental stress, we investigated the extracellular-matrix-associated macromolecules in *N. gypsea* biofilms *(*Figure 2*).* The eDNA content in *N. gypsea* biofilms increased after a 5-day incubation (Figure 2A; *p* < 0.05). Similarly, we observed a 2-fold increase in extracellular protein (Figure 2B, *p* < 0.0001) and polysaccharide (Figure 2C, *p* < 0.001) content between the 1- and 5-day incubation periods. These findings suggest that *N. gypsea* biofilms accumulate considerable extracellular macromolecular content, which may be crucial for the survival and infection of this filamentous fungus.

### 3.4. Scanning Electron Microscopy of N. gypsea Showing Cells Adhering and Forming Biofilms In Vitro

**Figure 3:**
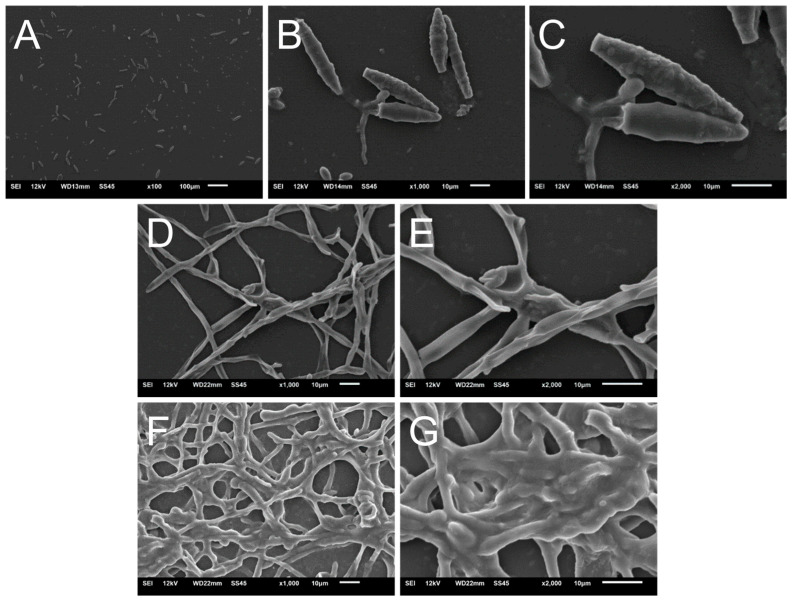
Scanning electron microscopy (SEM) of *N. gypsea* biofilm in vitro. Images were taken after 4 h (**A**) 100× (rectangle indicates the magnified region in (**B**,**C**)), (**B**) 1000×, and (**C**) [2000×; arrow indicates conidial tip germination], 1 day [(**D**) 1000× and (**E**) 2000×], and 5 days [(**F**) 1000× and (**G**) 2000×] of biofilm formation, denoting the exopolymeric matrix.

We visualized *N. gypsea* cell adhesion and biofilm formation in vitro using scanning electron microscopy (Figure 3). SEM images taken for 4 h post-adhesion revealed an abundance of macroconidia across the observed field (Figure 3A). Higher magnification images showed several elongated macroconidia and conidial tip germination (arrow; Figure 3B,C). In addition, septations and roughness on the conidia are visible (Figure 3C), showcasing the micromorphological characteristics of *N. gypsea*. Hyphal formation is visible on the plastic substrate 1-day post-incubation (Figure 3D,E), and a dense or thickening mycelial network, consisting of intertwined hyphae, predominates after 5 days of incubation (Figure 3F,G), denoting the exopolymeric matrix. These images demonstrate that *N. gypsea* biofilm formation progresses in an orderly fashion, including adhesion, germination, hyphal formation, and mycelium, which consists of a dense network of mature hyphae.

### 3.5. N. gypsea Heavily Colonizes and Forms Biofilm on Hair Ex Vivo

**Figure 4:**
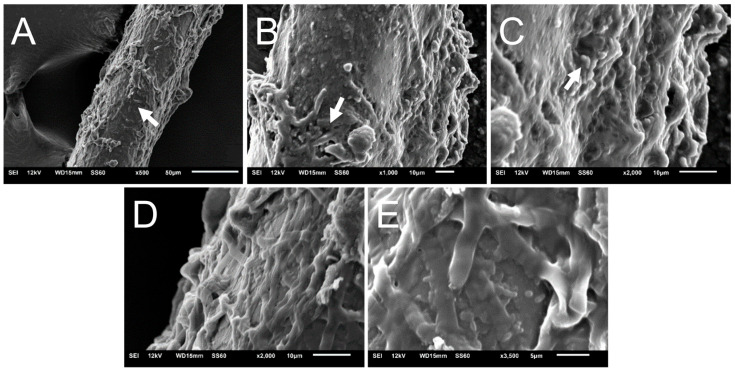
SEM of human hair infection by *N. gypsea* ex vivo. Infected hair is shown at a magnification of (**A**) 500×, (**B**) 1000× (arrows denote cracks in the hair fiber structure), (**C**) 2000×, (**D**) 2000×, and (**E**) 3500×—an extensive network of hyphae and exopolymeric matrix. White arrows in (**A**–**C**) indicate cracks in the hair follicle structure.

We investigated how *N. gypsea* infected human beard hair ex vivo using SEM (Figure 4). SEM images taken 5 days post-infection demonstrated complete colonization of and biofilm formation in the hair fiber (Figure 4A). The fungus adhered to the hair fiber and exhibited an abundant extracellular matrix (Figure 4B–E), consistent with the in vitro experiments. Furthermore, cracks in the hair structure are evident (arrow; Figure 4A–C), highlighting keratin degradation through ectothrix parasitism—a characteristic of this dermatophyte that has been well described. Our findings suggest that *N. gypsea* colonizes and forms strong biofilms in hair fibers, which may facilitate damage to keratinized surfaces.

### 3.6. Assessment of Nucleic Acid Integrity and Purity

The RNA integrity and purity of planktonic and biofilm forms were assessed using capillary and gel electrophoresis. Both samples exhibited high RNA concentration and purity, with characteristic bands of the 18S and 28S regions in capillary electrophoresis (Figure S2A,B). Additionally, agarose gel electrophoresis confirmed the results obtained through the in silico PCR tool, indicating that the sizes of the amplified fragments, in base pairs, for the GAPDH, *Sub7*, *Mmp2*, and *Mate2* genes were 89, 129, 107, and 125 bp, respectively (Table S1 and Figure S3).

### 3.7. N. gypsea-Biofilm-Derived Cells Showed Higher Virulence Factor Expression than Planktonic Cells

**Figure 5:**
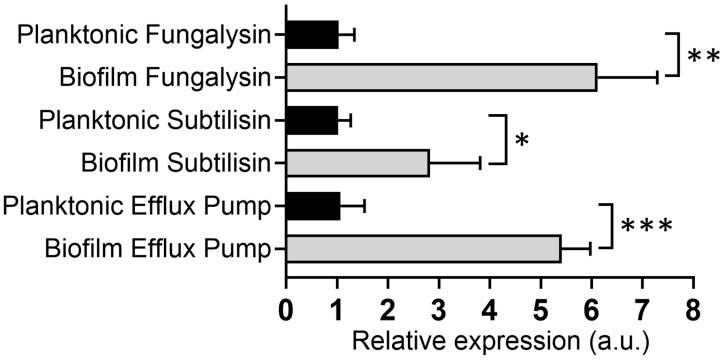
Relative expression of subtilisin, fungalysin, and efflux pump genes in *N. gypsea* planktonic and biofilm-derived cells after maturation of biofilm. Bars represent the average for three independent experiments. Error bars denote standard deviation. Significance (*** *p* < 0.001; ** *p* < 0.01; * *p* < 0.05) was determined using a paired two-tailed Student’s *t*-test. Relative expression a.u. indicates arbitrary units. This experiment was conducted multiple times, yielding similar results each time.

We compared the differences in *N. gypsea* virulence factor expression between biofilm-derived and planktonic cells, focusing on genes encoding proteolytic enzymes, including an extracellular metalloproteinase (*Mmp12*) and subtilisin 7 (*Sub7*). Additionally, we investigated the expression of the *Mate2* efflux pump gene, given its importance in antifungal resistance. *Mmp12* and *Sub7* were selected because they are considered virulence factors, given their ability to degrade keratin, which is of relevance in the pathogenesis of dermatophytosis [8]. *Mate2* was included due to the growing number of reports in the literature indicating a concerning rise in antifungal resistance [33,34,35]. When analyzing the relative expression of these genes, we observed a 5 (*p* < 0.01), 2 (*p* < 0.05), and 4 (*p* < 0.001)-fold increase in the expression of *Mmp12*, *Sub7*, and *Mate2*, respectively, of *N. gypsea*-biofilm-derived cells relative to planktonic cells (Figure 5). These results demonstrate that *N. gypsea* cells enclosed within biofilms highly express more virulence factors associated with dermatophytosis than single or individual planktonic cells.

## 4. Discussion

*N. gypsea* is a geophilic dermatophyte that has not been extensively studied, despite being isolated worldwide and causing infections in humans and animals, thus confirming the pathogenic potential of this filamentous fungus. Although *N. gypsea* biofilm formation has been previously described [16,36], no study has been conducted to analyze the extracellular matrix macromolecular content and important genes involved in this fungus’ pathogenesis. Furthermore, the medical mycology literature lacks data regarding the susceptibility of wild-type strains of *N. gypsea*. The strain used in this investigation demonstrated resistance to fluconazole, a second-generation azole, as compared to available data [37,38]. This finding highlights the increasing resistance to antifungal drugs commonly used in clinical practice. Previous studies have demonstrated that dermatophyte biofilms form in vitro within 3 days; however, variations between different species have also been reported [9,11,16,39,40]. *N. gypsea* exhibits slower kinetics compared to other dermatophytes. This behavior is evident in the XTT reduction assay and crystal violet staining results obtained in this study, which reached a plateau in metabolic activity and biomass during the 5-day incubation in vitro.

The extracellular matrix has a complex and variable composition depending on the fungal species. It consists mainly of macromolecules, including polysaccharides, proteins, lipids, extracellular DNA, and small molecules. To our knowledge, there was no characterization or quantification available on the macromolecular content of the biofilm extracellular matrix in dermatophytes. The most significant divergence in *N. gypsea* matrix macromolecular composition appears at the protein level; however, differences have also been observed in carbohydrate content, which has been the focus of most studies [41]. In our work, the total polysaccharide concentration in *N. gypsea* biofilm was higher than that of the other fungi studied. The composition of *Aspergillus fumigatus* carbohydrates is 43%, and proteins comprise 40% [41]. In contrast, the *Candida albicans* biofilm matrix consists of 25% polysaccharides, 55% proteins, and 5% nucleic acids [41]. Relative to these fungi, *N. gypsea* biofilms showed a much lower concentration of total proteins in the extracellular matrix. In contrast, the eDNA concentration of *N. gypsea* was higher than that found in *P. brasiliensis* and *C. albicans* biofilms [29,30]. Given the anionic nature of the eDNA present in biofilms, this nucleic acid can interact with certain classes of cationic drugs, such as allylamines (e.g., terbinafine) and azoles (e.g., fluconazole and ketoconazole) [42], thereby reducing their penetration into the biofilm. Consequently, fungal-biofilm-derived cells can adapt and become tolerant to these drugs, resulting in increased resistance to these therapeutics.

*N. gypsea* biofilms had significant biomass and substantial polysaccharide content in their extracellular matrix. Exopolysaccharides play an important structural and functional role in the development and maintenance of microbial biofilms. Recent studies have demonstrated the importance of these secreted exopolysaccharides as significant components of the extracellular matrix in *C. albicans* and *Aspergillus fumigatus* [41], playing a critical role in biofilm formation, drug resistance, and immune evasion [43].

The presence of the *N. gypsea* biofilm extracellular matrix was also visualized using scanning electron microscopy (SEM). The formation of hyphae and the extracellular matrix was demonstrated both in vitro and ex vivo. In the ex vivo model, cracks in the hair fiber structure were evident, highlighting keratin degradation through ectothrix parasitism, a characteristic of this dermatophyte that has been well described [44]. A coordinated network of hyphae was also observed, growing in all directions and forming a mycelium. Previous studies have demonstrated that *N. gypsea* biofilm formation is strain-dependent, with the majority of *N. gypsea* isolates only forming biofilms in the hair model, not in vitro [36]. Thus, considering that this study was conducted using only a single clinical isolate, the results may have limitations and may not fully reflect the variability and complexity of the biofilm formation process in this species. Therefore, our study contributes to a deeper understanding of this dermatophyte.

After adhering to the keratinized surface, the production of keratinolytic enzymes, which are related to virulence, is crucial for colonizing the surface and establishing the infection. Dermatophytes also secrete proteases in vivo, which are responsible for fungal colonization and the degradation of keratinized tissue during infection [17]. Dermatophyte-secreted endoproteases are numerous and belong to two large protein families [12], the subtilisins (serine proteases) and the fungalysins (metalloproteases). We used qPCR to investigate the presence of genes encoding fungalysins (*Mmp12*), subtilisin (*Sub7*), and efflux pumps (*Mate2*) in *N. gypsea* in both planktonic and biofilm forms. In addition to molecular methods, biochemical assays were conducted to evaluate the presence of key enzymatic factors associated with dermatophyte pathogenesis. The biochemical results obtained are consistent with the findings of Dukik et al. [45], indicating that the isolate utilized in this study possesses similar virulence factors. Furthermore, the isolate exhibited catalase activity—an enzymatic trait not previously reported in the genus *Nannizzia*, although it has been documented in other dermatophyte fungi, such as *Microsporum canis* [46]. Our results demonstrated a higher presence of these genes in mature biofilm-derived cells than in planktonic cells. These genes have been studied in planktonic cells of other dermatophytes such as *M. canis* and *T. rubrum* [17]. In this parasitic scenario, the keratinolytic process occurs through three consecutive steps: (i) deamination, (ii) sulfitolysis, and (iii) proteolysis, the latter being the step involving the proteases analyzed in the present study. Thus, dermatophytes can utilize complex keratinized structures as a source of carbon, nitrogen, and sulfur for their growth and persistence [47].

The *Mate2* encodes for an efflux pump, and its upregulation in *N. gypsea* biofilms suggests its potential involvement in antifungal drug resistance mechanisms. Although not fully characterized by the species *N. gypsea*, homologous efflux pump structures have already been found in other microorganisms, highlighting their possible role in the emergence of antimicrobial resistance [48]. Future studies should investigate and further explore the cellular mechanisms of *N. gypsea* biofilm that regulate the production and secretion of subtilisin and fungalysin into the extracellular environment, as well as the function of the Multidrug and Toxin Extrusion Protein 2 efflux pump. Additionally, comparisons between different strains of the microorganism should be established as this study is limited to using only one *N. gypsea* strain.

This pioneering study elucidates the structural characteristics of the biofilm formed by the dermatophyte *N. gypsea*, with an emphasis on its formation kinetics and the macromolecular composition of its extracellular matrix. In addition, the analysis of key virulence factors involved in the pathogenesis of these infections contributes to a deeper understanding of dermatophyte fungal biofilms. However, further studies employing alternative infection models, such as three-dimensional cell cultures and reconstructed skin models, are necessary to deepen our knowledge of host–pathogen interactions and to support the development of novel antifungal therapeutic strategies. Furthermore, even though biofilm formation by dermatophytes has been suggested in vivo models, there are no reports that we are aware of describing or confirming this important process. Therefore, the existence of dermatophyte biofilms in vivo remains a hypothesis that has not yet been experimentally confirmed, but it needs special attention to understand dermatophyte pathogenesis.

## Figures and Tables

**Table 1:** Primers used in the qPCR studies.

Gene	Sequence	GenBank
GAPDH	F 5′ GCCGTGTTGTTGACCTCATC 3′ R 5′ GCGATGTAGGCTGTGAGAGA 3′	NW_003345199.1
Subtilisin (*Sub7*)	F 5′ ACTGTCGCCGGTACCAAAT 3′ R 5′ GGCGTCGTTGGTAGCAAAT 3′	OM397965.1
Multidrug and Toxin Extrusion Protein 2 (*Mate2*)	F 5′ GCCATCACCCAGCTCTTCTA 3′ R 5′ CCCTTGCCGTTGTTGTTCAT 3′	NW_003345198.1
Extracellular elastolytic metalloproteinase (*Mmp12*)	F 5′ GCCGTCAGCTTAGCCAGTAT 3′ R 5′ AGGCCGACTAGCTTCTTGTT 3′	NW_003345200.1

**Table 2:** Biochemical tests were used to characterize the species and virulence factors of the clinical isolate used in these studies.

Biochemical Test	*Nannizzia gypsea* F35 LLH (Clinical Isolate)
Catalase	Positive
Lipase	Positive
Protease	Positive
Hemolytic activity	Negative
Urease	Positive

## Data Availability

The data presented in this study are available on request from the corresponding author. The data are not publicly available due to privacy restrictions.

## Supplementary Materials

The following supporting information can be downloaded at: https://www.mdpi.com/article/10.3390/jof11060455/s1, Figure S1.: Report generated by the MALDI Biotyper® Sirius (Bruker), showing the respective score values and matches; Figure S2.: Report on the analysis of the (A) planktonic and (B) biofilm RNA samples performed using the Bioanalyzer 2100 (Agilent Technologies); Figure S3.: Agarose gel electrophoresis (1.5%) of the amplification products for each gene. The gel was stained with SYBR Safe (Thermo Fisher Scientific); Table S1: Amplicon length of each gene analyzed by qPCR.
