# Supplementary material for: Characterization of Biofilm Formation by the Dermatophyte Nannizzia gypsea
Source: J Fungi (Basel). 2025 Jun 14;11(6):455. doi: 10.3390/jof11060455 (PMC12194254; doi:10.3390/jof11060455)
Supplement: Supplementary file 1 [file jof-11-00455-s001.zip › jof-3590875-supplementary.pdf]

Supplementary Figures and Table

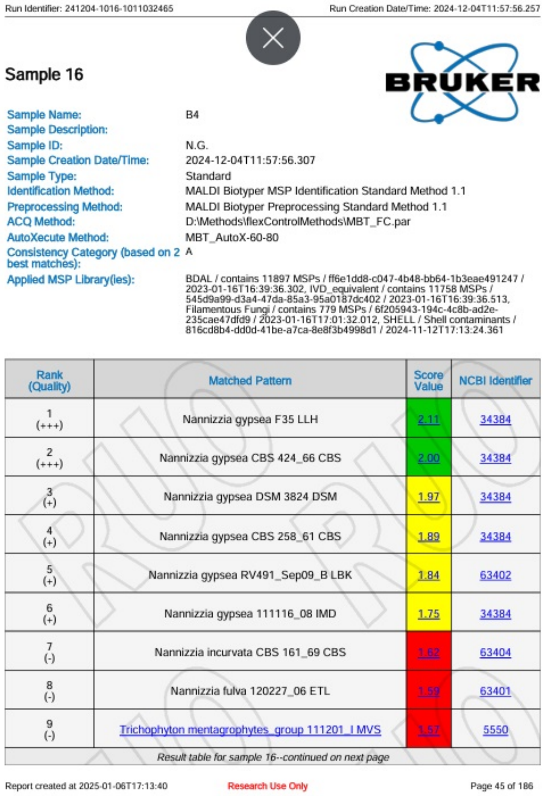

Run Identifier: 241204-1016-1011032465

Run Creation Date/Time: 2024-12-04T11:57:56.257

Sample 16

Sample Name: B4

Sample Description:

Sample ID: N.G.

Sample Creation Date/Time: 2024-12-04T11:57:56.307

Sample Type: Standard

Identification Method: MALDI Biotyper MSP Identification Standard Method 1.1

Preprocessing Method: MALDI Biotyper Preprocessing Standard Method 1.1

ACQ Method: D:\Methods\flexControlMethods\MBT_FC.par

AutoXecute Method: MBT_AutoX-60-80

Consistency Category (based on 2 best matches): A

Applied MSP Library(ies): BDAL / contains 11897 MSPs / ff6e1dd8-c047-4b48-bb64-1b3eae491247 / 2023-01-16T16:39:36.302, IVD_equivalent / contains 11758 MSPs / 545d9a99-d3a4-47da-85a3-95a0187dc402 / 2023-01-16T16:39:36.513, Filamentous Fungi / contains 779 MSPs / 6f205943-194c-4c8b-ad2e-235cae47dfd9 / 2023-01-16T17:01:32.012, SHELL / Shell contaminants / 816cd8b4-dd0d-41be-a7ca-8e8f3b4998d1 / 2024-11-12T17:13:24.361

Rank (Quality)	Matched Pattern	Score Value	NCBI Identifier
1 (+++)	Nannizzia gypsea F35 LLH	2.11	34384
2 (+++)	Nannizzia gypsea CBS 424_66 CBS	2.00	34384
3 (+)	Nannizzia gypsea DSM 3824 DSM	1.97	34384
4 (+)	Nannizzia gypsea CBS 258_61 CBS	1.89	34384
5 (+)	Nannizzia gypsea RV491_Sep09_B LBK	1.84	63402
6 (+)	Nannizzia gypsea 111116_08 IMD	1.75	34384
7 (-)	Nannizzia incurvata CBS 161_69 CBS	1.62	63404
8 (-)	Nannizzia fulva 120227_06 ETL	1.59	63401
9 (-)	Trichophyton mentagrophytes group 111201_I MVS	1.57	5550

Result table for sample 16--continued on next page

Report created at 2025-01-06T17:13:40 Research Use Only Page 45 of 186

Figure S1. Report generated by the MALDI Biotyper® Sirius (Bruker), showing the respective score values and matches.

A

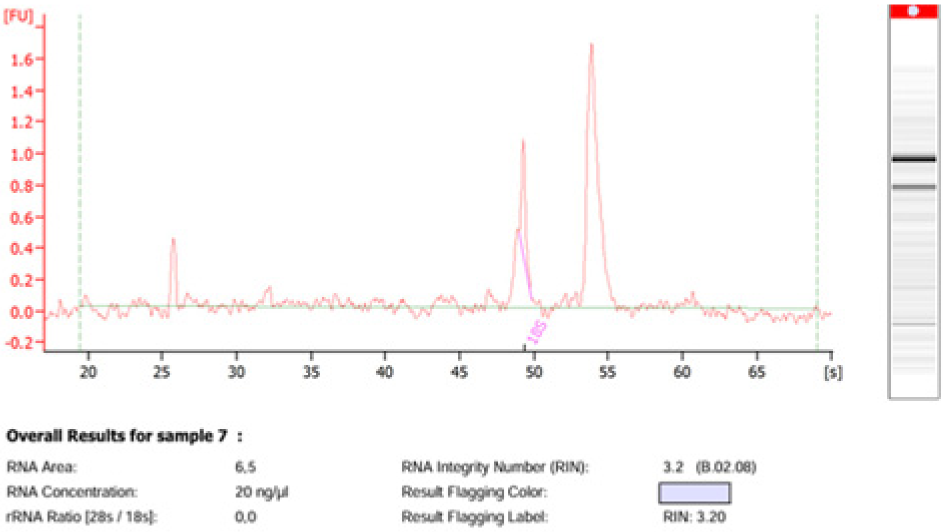

B

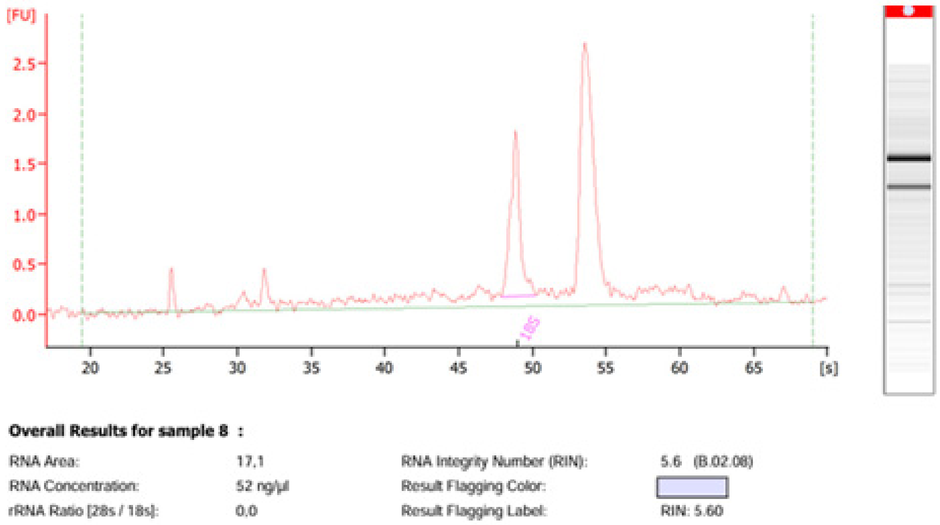

Figure S2. Report on the analysis of the **(A)** planktonic and **(B)** biofilm RNA samples performed using the Bioanalyzer 2100 (Agilent Technologies).

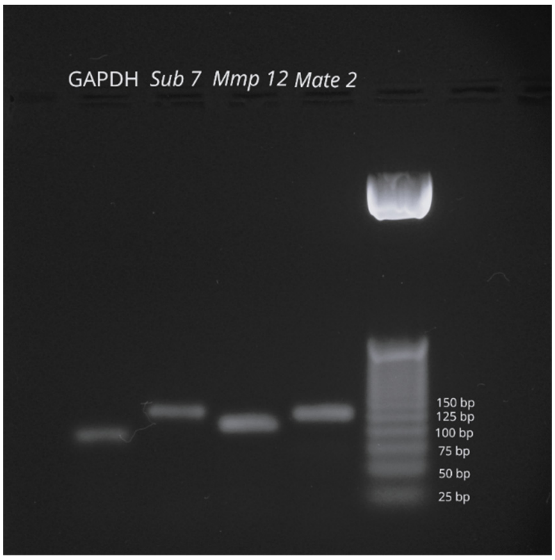

Figure S3. Agarose gel electrophoresis (1.5%) of the amplification products for each gene. The gel was stained with SYBR Safe (Thermo Fisher Scientific)

Supplementary Table

Table S1. Amplicon length of each gene analyzed by qPCR.

Gene	Length (bp)
GAPDH	89
Sub7	129
Mmp12	107
Mate2	125
